# Xiaoyaosan Exerts Therapeutic Effects on the Colon of Chronic Restraint Stress Model Rats via the Regulation of Immunoinflammatory Activation Induced by the TLR4/NLRP3 Inflammasome Signaling Pathway

**DOI:** 10.1155/2021/6673538

**Published:** 2021-01-07

**Authors:** Hui-Zheng Zhu, Yu-Dan Liang, Wen-Zhi Hao, Qing-Yu Ma, Xiao-Juan Li, Yu-Ming Li, Jia-Xu Chen

**Affiliations:** ^1^Jiangmen Central Hospital, Affiliated Jiangmen Hospital of Sun Yat-Sen University, Jiangmen 529030, China; ^2^Formula-Pattern Research Center, School of Traditional Chinese Medicine, Jinan University, Guangzhou 510632, Guangdong, China; ^3^The First Affiliated Hospital, Jinan University, Guangzhou 510632, Guangdong, China; ^4^Integrated Chinese and Western Medicine Postdoctoral Research Station, Jinan University, Guangzhou 510632, China

## Abstract

Depression is the neurological manifestation most commonly associated with gastrointestinal diseases. The release of inflammatory cytokines mediated by TLR4/NLRP3 inflammasome signaling-induced immunoinflammatory activation may represent a common pathogenic process underlying the development of gastrointestinal diseases and depression. Clinical studies have indicated that Xiaoyaosan (XYS) can relieve depressive behavior by improving gastrointestinal symptoms. We previously demonstrated that XYS can reduce colonic inflammation in a rat model of chronic unpredictable mild stress; however, the precise anti-inflammatory mechanisms involved remain unclear. Here, we investigated whether XYS can ameliorate depressive behavior through regulating the TLR4/NLRP3 inflammasome signaling pathway, thereby inhibiting immunoinflammatory activation and reducing colonic proinflammatory cytokine levels. Fifty-two healthy male Sprague–Dawley rats were randomly divided into four groups (control, model, XYS, and fluoxetine). The latter three groups were subjected to 21 days of chronic restraint stress to generate a model of stress-induced depression. XYS and fluoxetine were administered intragastrically. Behavioral changes in the rats were assessed after 21 days. Serum and colon samples were collected, and the relative levels of the inflammation indicators IL-6, IL-1*β*, and TNF-*α* were determined by ELISA. Pathological changes in colon tissue were assessed by hematoxylin and eosin staining. The levels of TLR4, MyD88, NF-*κ*B-p65, TAK1, IRAK1, and TRAF6 were detected by immunohistochemistry, while the gene and protein expression levels of TLR4, MyD88, NF-*κ*B-p65, TAK1, IRAK1, TRAF6, NLRP3, ASC, and caspase-1 were detected by quantitative polymerase chain reaction (qPCR) and Western blotting. The results indicated that XYS could improve the depressive-like behavior and the weight loss of rats with stress-induced depression. Furthermore, depressed rats treated with XYS exhibited decreased expression levels of TLR4, MyD88, NF-*κ*B-p65, TAK1, IRAK1, TRAF6, NLRP3, ASC, and caspase-1 in colonic tissue; reduced colon and serum concentrations of the inflammatory factors IL-6, IL-1*β*, and TNF-*α*; and lowered levels of colonic inflammation.

## 1. Introduction

Depression is the leading cause of disability worldwide and a major contributor to the overall global disease burden. According to a recent World Health Organization (WHO) report, between 2005 and 2015, 322 million people suffered from depression worldwide and the number of affected patients increased by 18.4% [1]. The main clinical feature of these patients is significant and persistent depression, which may be associated with a high incidence of comorbid somatic symptoms such as gastrointestinal motility disorder and insomnia. Depression can impair the function of certain parts of the gastrointestinal tract, thereby increasing the risk of gastrointestinal complications. Furthermore, depression is the neurological manifestation that is most commonly associated with gastrointestinal diseases, such as irritable bowel syndrome and inflammatory bowel disease [2, 3]. Indeed, several studies have shown that the prevalence of mental disorders in patients with gastrointestinal symptoms is approximately 60–85% [4–6].

It has been suggested that depression may represent a chronic low-level inflammatory response involving neuroimmune-endocrine factors [7]. An increasing number of studies have found that specific depressive symptoms, particularly somatic symptoms, are associated with changes in inflammation-related immune system components [8]. Human immunity is divided into natural (innate) and adaptive immunity. The former occurs via pattern recognition receptors (PRRs), which recognize highly conserved pathogen-associated molecular patterns (PAMPs) and produce corresponding immune responses. Three types of PRRs have been identified, namely, Toll-like receptors (TLRs), NOD-like receptors (NLRs), and C-type lectin receptors (CLRs) [9]; of these, TLR4 and the inflammasome-associated NOD-like receptor pyrin domain containing 3 (NLRP3) are most closely linked to depression.

Studies have indicated that TLR4 activation and the inflammatory factors it mediates are closely related to several depressive symptoms and may be directly linked to their occurrence and development [10]. Long-term chronic stress can activate NLRP3, producing a corresponding inflammatory response that contributes to the pathogenesis of depression [11, 12]. TLRs are important mediators of innate immune responses in the intestinal mucosa and play an important role in maintaining microecological homeostasis in the intestinal tract [13]. Recent studies have shown that NLRP3 is involved in maintaining the stability of the intestinal environment and is also closely related to the occurrence and development of inflammatory bowel disease [14].

In humans, the immune system is the first line of defense against both exogenous and endogenous threats, responding rapidly to attack through the regulation of the levels of both proinflammatory and anti-inflammatory factors. The accumulation of inflammatory cytokines can lead to depression, as well as other psychiatric and gastrointestinal diseases, and reducing the levels of these factors can show good therapeutic effects. In summary, the release of inflammatory cytokines mediated by TLR4/NLRP3 inflammasome signaling pathway-induced immunoinflammation may represent a common pathogenic mechanism underlying the development of gastrointestinal diseases and depression.

Xiaoyaosan (XYS), a classic agent used in traditional Chinese medicine (TCM), was first described by the “Taiping Huimin Heji Jufang” of the Song Dynasty (960–1127 AD). XYS is a decoction composed of the following eight commonly used Chinese herbs: Radix Angelicae Sinensis, Radix Paeoniae Alba, Poria, Radix Bupleuri, Radix Glycyrrhizae, Rhizoma Atractylodis Macrocephalae, Herba Menthae, and Rhizoma Zingiberis Recens. XYS has been used in clinical practice for more than 1,000 years in China and is commonly used to treat diseases such as depression, functional dyspepsia, hepatitis, cirrhosis, gastroduodenal ulcer, chronic gastritis, premenstrual tension, and perimenopausal syndrome [15]. Pharmacological studies have shown that XYS can protect the liver, enhance gastrointestinal peristalsis, regulate the central nervous system (CNS) and endocrine function, and help resist stress [16]. A recent analysis of the use of antidepressants indicated that more than 55% of the Chinese medicines used for treating depression targeted the spleen-stomach meridian [17]. For instance, the Radix Acanthopanacis Senticosi capsule can reportedly relieve colitis and improve depressive symptoms [18]. As depression has been reported to induce injury in the colon through oxidative stress and inflammation, we chose depression-induced colitis as a target in this study [18]. We have previously demonstrated that XYS treatment can reduce colonic inflammation in a rat model of chronic unpredictable mild stress (CUMS) [19]; however, the underlying anti-inflammatory mechanism remains unclear. In this study, we hypothesized that XYS could improve depressive behavior by regulating the TLR4/NLRP3 inflammasome signaling pathway, thereby inhibiting immunoinflammatory activation and, consequently, reducing the levels of inflammatory cytokines in the colon.

## 2. Materials and Methods

### 2.1. Animals and Grouping

Healthy, specific pathogen-free male Sprague–Dawley rats (license no. SCXK (YUE) 2013-0001) weighing 200 ± 20 g were purchased from the Experimental Animal Center of Guangzhou University of Traditional Chinese Medicine. The rats were maintained in plastic cages at a room temperature of 22 ± 2°C and a relative humidity of 35 ± 5%. The rats were allowed to drink deionized water and eat conventional food ad libitum. After 7 days of adaptive feeding, the rats were randomly divided into 4 groups: a control group, a chronic restraint stress model group, an XYS treatment group, and a fluoxetine treatment group, with 13 rats allocated to each group. The animal experiments were approved by the Ethics Committee of the Guangzhou University of Traditional Chinese Medicine (ethical protocol no. 20180829001) and conformed with the guidelines of the National Institutes of Health for the Care and Use of Animals.

### 2.2. Drug Preparation and Intervention

The XYS prescription consisted of eight herbal medicines, as shown in Table 1. XYS Chinese herbal pieces were provided by Beijing Tongrentang (Bozhou) Pieces Co., Ltd. (Bozhou, China). The XYS dry powder was prepared by Jiuzhitang Co., Ltd. (Changsha, China) in accordance with the process described in the 2015 edition of the “Chinese Pharmacopoeia” [20]. The Xiaoyaosan dry powder was prepared by mixing 100 g of *Bupleurum chinense*, 100 g of *Paeonia lactiflora*, 100 g of *Angelica sinensis*, 100 g of fried *Atractylodes macrocephala*, 100 g of *Poria cocos*, 80 g of *Glycyrrhiza uralensis*, 20 g of mint, and 100 g of ginger. We previously detected eight compounds in XYS by high-performance liquid chromatography–mass spectrometry (HPLC–MS/MS), and the results suggested that the eight compounds might be XYS quality control references [21]. The fluoxetine hydrochloride tablets used in this experiment were purchased from Patheon (France) and packaged by Lilly Suzhou Pharmaceutical Co., Ltd. (20 mg/tablet, Suzhou, China).

Treatments were administered orally to the rats in each group 30–60 min before modeling. The drug dosage was determined according to the mean adult bodyweight (at 60 kg/day). The volume of the gavage solution was 1 mL/100 g of bodyweight. Fluoxetine was administered intragastrically at a dose of 2 mg/(kg·day^−1^). The XYS dosage was 2.224 g/(kg day^−1^). The control group and the model group received an equal volume of deionized water.

### 2.3. Quality Control of XYS

**Figure 1:**
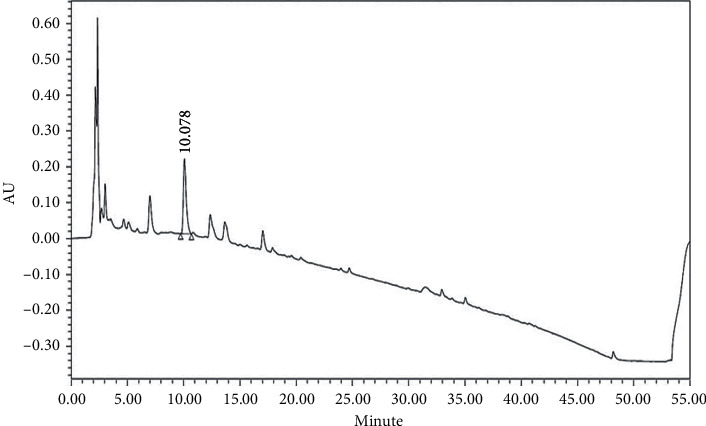
Quality control of Xiaoyaosan (quantitative paeoniflorin analysis).

XYS quality control was carried out according to the 2015 edition of the Chinese Pharmacopoeia [20]. As the pharmacopoeia standard states that 1 g of XYS should contain at least 4 mg of paeoniflorin, HPLC was used to detect the content of paeoniflorin in XYS as follows: 1 g of XYS was put into 25 mL of 70% methanol, sonicated for 30 min, and filtered through a 0.22 *μ*m filter. The chromatographic column used was an Agilent ZORBAX Eclipse XDB C-18 (250 × 4.6 mm, 5 *μ*m), and the column temperature was set at 350°C. The chromatographic conditions were A: 0–5 min 0.1%–70% methanol, 5–40 min 70%–10%, 40–45 min 10%–0%, and 40–50 min 0%; B: 0–5 min 0.1%–30% methanol, 5–40 min 30%–90%, 40–45 min 90%–100%, and 40–50 min 100%. The detection wavelength was 230 nm, the injection volume was 10 *μ*L, and the flow rate was 1 mL/min. The paeoniflorin content was 5.3299 mg/g, and the retention time was 10.078 min, which conformed to the quality standard stipulated in the Pharmacopoeia (Figure 1).

### 2.4. Chronic Immobilization Stress (CIS) Modeling Procedure

Chronic restraint stress modeling was performed as previously described [22]. Rats in the model, XYS, and fluoxetine groups were bound to a special restraint frame. Two soft bands were attached to the chest and abdomen of each rat. The rats were then placed in a feeding box for 3 h/day. The restraint time was random, and the procedure was performed for 21 consecutive days. Rats in the normal group were fed in their respective feeding boxes for 21 consecutive days.

### 2.5. Bodyweight, Sugar Preference Test, Open-Field Test, and Forced Swim Test

The bodyweight of the rats in each group was determined at 08:00 every day using an electronic scale, and the mean weight of the rats in each group on days 0, 7, 14, and 21 was calculated and compared.

The analysis of sugar consumption was carried out on days 0 and 21 of modeling. Before the experiment, the rats in each group were fed in a single cage. Two bottles containing a 1% sucrose solution were placed in each cage, and the rats were allowed to drink freely for 24 h. Next, one bottle containing a 1% sucrose solution and one containing pure water were placed in each cage, and the rats were allowed to drink freely for 24 h. Then, the rats were deprived of food and water for 24 h. During the experiment, one bottle containing a 1% sucrose solution and one containing pure water were placed in each cage, and the rats were allowed to drink freely for 3 h. The weight of the sucrose solution and the pure water before and after the experiment was then recorded. The rate of sugar water consumption was calculated as sugar water consumption/total liquid consumption×100%. This experiment was carried out to evaluate the degree of loss of pleasure.

The open-field test (OFT) was performed on days 0 and 21 of the experiment. An open-field box was placed in the middle of the test room, and a camera was placed above the center grid and connected to a computer. Measurements were taken under quiet conditions. Before the experiment, rats were placed in the dark environment of the test room for 10 min to acclimatize. The operator then grasped the tail root of a rat and placed the rat in the middle compartment of the open-field box. Recording and timing were then synchronized. The time of residence in the central grid, the total number of passes, and the number of entries into the central zone were recorded within a 5 min period. After 5 min, the rats were removed, and the bottom of the box was wiped thoroughly with a towel dipped in clean water containing a low concentration of alcohol. The next rat was then observed.

The device for the forced swim test (FST) consisted of a transparent acrylamide resin barrel 40 cm high and 30 cm in diameter. The rats were placed in the barrel with water (23–25°C) to a depth of 32 cm to ensure that their hind limbs could not touch the bottom of the barrel. On the first day of the experiment, all the rats were subjected to a 15 min swim, dried, and then allowed to continue feeding. After 24 h, the forced swimming test was conducted for 5 min, and the immobility time (characterized as floating on the water surface, immobility, or making small movements) was recorded and analyzed. After each test, the barrel was filled with clean water to avoid cross fecal and olfactory interference.

### 2.6. Sample Collection

All the animals were euthanized on day 21 (after behavioral testing). Then, 1 cm of colon tissue (10 cm from the anus) was removed and immediately rinsed with normal saline and fixed in 10% formaldehyde. The colon tissue was then embedded in paraffin wax, sectioned to a thickness of 4 *μ*m, and the sections were placed on poly-lysine-coated glass slides. Two other 1 cm colonic tissue samples were placed in an EP tube and stored at −80°C for later analysis. Blood samples (2-3 mL) were collected from the abdominal aorta, left at room temperature for 10–20 min, and then centrifuged at 3,500× *g* for 20 min at 4°C. The serum collected after centrifugation was then filtered, sterilized, inactivated, and stored at −20°C for subsequent analysis.

### 2.7. ELISA Analysis

The colon and serum levels of IL-1*β* (JER-01), TNF-*α* (JER-06), and IL-6 (JER-04) were determined using commercial ELISA kits (Joyee Biotechnics Co., Ltd., Shanghai, China) according to the manufacturer's instructions. Absorbance was detected at 450 nm using a microplate reader (BioRad, USA). Finally, a standard curve was generated to determine the concentration of each factor.

### 2.8. Hematoxylin and Eosin Staining

Colonic tissue samples were fixed in a 10% formalin solution, decalcified, dehydrated, made transparent, and finally embedded in paraffin. Then, 5 *μ*m-thick tissue sample sections were prepared using a microtome. The sections were dewaxed with xylene, passed through an ethanol series to water, and finally stained with hematoxylin and eosin (H&E). The results were assessed under a microscope.

### 2.9. Immunohistochemical Analysis

 Immunohistochemistry was used to determine the localization of TLR4, MyD88, NF-*κ*B-p65, TAK1, IRAK1, and TRAF6 in colonic tissue. Colonic tissue samples were fixed in 4% paraformaldehyde for 30–60 min, washed with phosphate-buffered saline (PBS) twice for 2 min, and dehydrated at 5°C. The tissues were then embedded in paraffin, sliced into 4 *μ*m-thick sections, and the sections were placed onto glass slides. Endogenous enzymes were inactivated using 3% hydrogen peroxide (H_2_O_2_) after routine dewaxing to water. The sections were then incubated with 0.01 mol/L citrate for antigen retrieval and blocked in a 3% bovine serum albumin (BSA) solution for 30 min. Subsequently, the sections were incubated with primary antibodies targeting TLR4 (Zen, 1 : 100), MyD88 (Zen, 1 : 100), NF-*κ*B-p65 (Zen, 1 : 100), TAK1 (Zen, 1 : 100), IRAK1 (PTG, 1 : 250), and TRAF6 (Zen, 1 : 100) overnight at 4°C in a humid box. The sections were then slightly dried and incubated with an HRP-conjugated secondary antibody against the corresponding species and genera at room temperature for 50 min. The slides were then washed four times with PBS, 5 min each wash, followed by DAB and nuclear staining. Finally, the slides were washed in distilled water, dehydrated, made transparent, and sealed with resin. The sections were observed under a microscope. Cells with brown granules were classified as positively stained. Immunohistochemical staining integrated optical density (IOD) was analyzed using Image-Pro Plus 6.0 (Media Cybernetics, Inc., Rockville, MD, USA). At least three random sections from each group were analyzed, and 200 fields of view were imaged whilst ensuring that the background lighting was consistent. Image-Pro Plus 6.0 software was then used to select the same brown-yellow color as a unified criterion for judging the positivity on all photographs. The positive IOD of each image was then determined.

### 2.10. RNA Isolation and RT-qPCR Analysis

First, 20–50 mg of tissue was placed into a mortar and quickly ground with a small amount of liquid nitrogen. Once the tissue had softened, more liquid nitrogen was added, and the procedure was repeated three times. Then, 1 mL of TRIzol was added, and the sample was transferred to a centrifuge tube and left at room temperature for 5 min. The sample was then centrifuged at 12,000 ×g for 10 min at 4°C. The supernatant was removed, 200 *μ*L of chloroform was added, and the tube was capped, followed by vigorous shaking for 15 s. The sample was left at room temperature for 5 min and then centrifuged at 12,000 ×g for 15 min at 4°C. The colorless upper water phase was then removed (400 *μ*L) and added to 500 *μ*L of isopropanol. The tube was capped, and the contents were mixed by inversion 10 times; the tube was then left at room temperature for 10 min. Samples were then centrifuged at 12,000 ×g for 10 min at 4°C. The supernatant was discarded, and 1 mL of 75% ethanol was added. The sample was then washed and centrifuged at 7,500 ×g for 5 min at 4°C. The supernatant was removed and the sample air-dried. Finally, the RNA was dissolved in 30 *μ*L of RNase-free water. Then, 4 *μ*L of RNA was diluted 25-fold and used to determine the RNA concentration and purity.

qPCR was performed using the Brilliant III Ultra-Fast SYBR Green QPCR Master Mix Kit (Agilent Technologies) in a Stratagene Mx3000P Real-Time PCR System (Agilent Technologies). The reaction mixture included 10 *μ*L of 2x SYBR Green QPCR Master Mix, 3.7 *μ*L of RNase-free water, 0.3 *μ*L of control fluorescent dye, 2 *μ*L of each forward and reverse primer, and 2 *μ*L of cDNA. The primer sequences (Generay Biotechnology Company, Shanghai, China) are listed in Table 2. The reaction conditions were as follows: an initial denaturation of 95°C for 3 min, followed by 40 cycles of denaturing at 95°C for 5 s and annealing at 60°C for 1 min. After cycling, a dissolution curve was measured under the following conditions: 95°C for 15 s, 55 °C for 1 min, and 95°C for 30 s. During heating from 55°C to 95°C, fluorescence was measured at each 0.5°C step. The amplification efficiency of the primers was measured using a serially diluted cDNA template. qPCR was performed for samples from each group. Reaction specificity was evaluated by analyzing the dissolution curve, and quantitative analysis of the fluorescence of the PCR products was carried out using the 2^−ΔΔCT^ method.

### 2.11. Western Blot Analysis

The levels of TLR4, MyD88, NF-*κ*B-p65, TAK1, IRAK1, TRAF6, NLRP3, ASC, and caspase-1 were quantified by Western blotting. First, proteins were extracted by cutting and grinding tissue samples in RIPA lysis buffer. The lysates were then centrifuged, and the supernatants were collected. Protein concentrations were determined using a BCA protein quantitation kit (GeneCopoeia). Proteins were then separated by SDS–PAGE and subsequently transferred onto methanol-pretreated PVDF membranes (Millipore Corporation, USA). The membranes were then blocked with 5% skimmed milk powder at room temperature for 1 h and incubated with the following antibodies: anti-TLR4 rabbit pAb (Zen, 1 : 1,000); anti-TRAF6 rabbit pAb (Zen, 1 : 1,000); anti-TAK1 rabbit pAb (Zen, 1 : 1,000); anti-NF-*κ*B-p65 mAb (Zen, 1 : 1,000); anti-MYD88 rabbit pAb (Zen, 1 : 800); anti-IRAK1 (PTG, 1 : 1,000); anti-ASC (Bioss, 1 : 1,000); anti-NLRP3 (Bioss, 1 : 1,500); and anti-caspase-1 (Bioss, 1 : 800). The membranes were subsequently incubated with the appropriate secondary antibodies for 1 h (HRP-conjugated goat anti-rabbit IgG (Ubio, 1 : 5,000) and HRP-conjugated goat anti-mouse IgG (Ubio, 1 : 5,000)). GAPDH and *β*-tubulin (Ubio) were used as reference proteins.

### 2.12. Statistical Analysis

Data are expressed as means ± standard deviation (SD). SPSS 25.0 (SPSS Inc., Chicago, IL, USA) was used to test the data for normality and homogeneity of variance. Repeated measurement data were first analyzed by repeated-measures analysis of variance (ANOVA). The remaining data were analyzed using one-way ANOVA. The least significant difference (LSD) method was used for comparison. The artwork was created using GraphPad Prism 7.0 (GraphPad Software, La Jolla, CA, USA).

## 3. Results

### 3.1. The Effect of XYS on the Bodyweight of Rats with CIS

**Figure 2:**
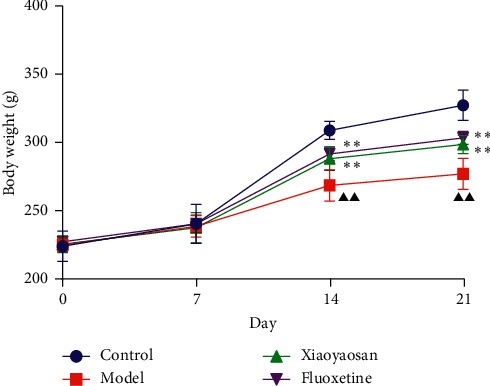
The bodyweight for each group. Data are expressed as means ± SD, *n* = 8. *p* < 0.01, compared with the normal group; ^*∗∗*^*p* < 0.01, compared with the model group.

On days 0 and 7, no difference in bodyweight was observed across the four groups. However, from day 14, the bodyweight of the XYS and fluoxetine groups showed a significant increase when compared with the model group; this difference was also evident on day 21 (day 14: *p* < 0.01, *F* = 31.471; day 21: *p* < 0.01, *F* = 70.129). The results showed that, from day 14, chronic stress led to significant weight loss in the three groups. However, compared with the model group, XYS and fluoxetine treatments alleviated the weight loss (Figure 2).

### 3.2. The Effect of XYS on the Depressive-Like Behavior of Rats with CIS

**Figure 3:**
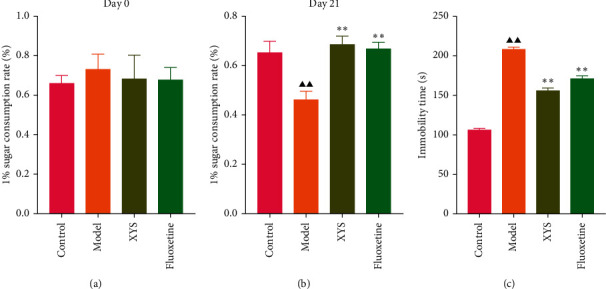
The sugar consumption rate (a)-(b) and forced swimming test (c). Data are expressed as means ± SD, *n* = 8. ^▲▲^*p* < 0.01, compared with the control group; ^*∗∗*^*p* < 0.01, compared with the model group. XYS, Xiaoyaosan.

**Figure 4:**
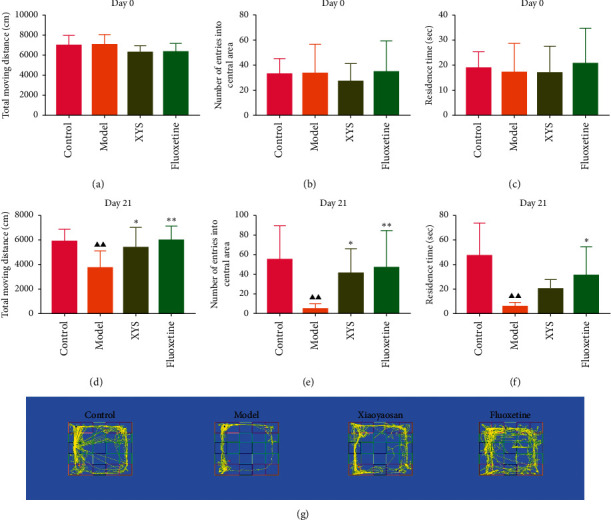
Changes in the depressive-like behaviors of rats with chronic stress-induced depression. The total distance traveled by rats in each group at day 0 (a); the total distance traveled by rats in each group on day 21 (d); the number of entries into the central area by rats in each group on day 0 (b); the number of entries into the central area by rats in each group on day 21 (e); the residence time of rats in each group on day 0 (c); and the residence time of rats in each group on day 21 (f). Data are expressed as means ± SD (*n* = 8), ^*∗*^*p* < 0.05 and ^*∗∗*^*p* < 0.01 vs. the model group; ^▲^*p* < 0.05 and ^▲▲^*p* < 0.01 vs. the control group. XYS, Xiaoyaosan. (g) Representative movement trails of rats in each group on day 21 as assessed by video tracking software.

To evaluate depressive-like behaviors, we carried out several behavioral tests, including a sucrose preference test (SPT), an OFT, and a FST. On day 0, the sugar preference rate was not significantly different across the four groups (*p*=0.396, *F* = 1.027). However, on day 21, the consumption rate in the model group was significantly lower than that of the XYS, fluoxetine, and control groups (*p* < 0.01, *F* = 51.385), suggesting that the animals in the model group were experiencing a marked loss of pleasure. This indicated that the model had been successfully established (Figures 3(a) and 3(b)). The results of the FST showed that the immobility time of the rats in the depression model group was significantly increased (*p* < 0.01, *F* = 35.389), whereas that of the rats in the XYS and fluoxetine groups was decreased (all *p* < 0.01, *F* = 35.389) (Figure 3(c)). In the OFT, the total distance traveled in 5 min by the rats in the model group (*p* < 0.01, *F* = 5.693), as well as the number of times they entered the central area (*p* < 0.01, *F* = 4.931), was significantly reduced compared with that in the control group. However, compared with the model group, the total distance traveled in 5 min and the number of times the rats entered the central area increased after treatment with XYS (*p* < 0.05, *F* = 4.931) and fluoxetine (*p* < 0.01, *F* = 4.931). Fluoxetine treatment also increased the residence time in the central area (*p*=0.01, *F* = 7.455) (Figure 4). Representative movement trails of the rats in each group during the OFT on day 21 are shown in Figure 4.

### 3.3. The Effect of XYS on Colon Pathology

**Figure 5:**
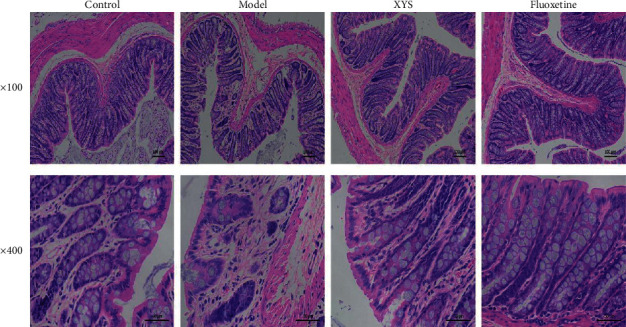
The effect of Xiaoyaosan (XYS) on colon pathology. Hematoxylin and eosin staining of the colon (×100 magnification, ×400 magnification).

As shown in Figure 5, the colon mucosa of the control group was intact, the epithelial cells were orderly arranged, no infiltration of inflammatory cells was seen, and the glands of the lamina propria were clear, well-arranged, and rich in goblet cells. In the model group, the colon mucosa was obviously absent, the glands in the lamina propria were damaged or absent, there were fewer goblet cells, and a large number of inflammatory cells were infiltrated. However, compared with the model group, treatment with XYS or fluoxetine could prevent epithelial hyperplasia around the colon mucosa, reduce the loss of goblet cells, decrease the infiltration of inflammatory cells, and restore basic goblet cell morphology.

### 3.4. The Effect of XYS on the Localization of TLR4, MyD88, NF-*κ*B-p65, TAK1, IRAK1, and TRAF6 in the Colon

**Figure 6:**
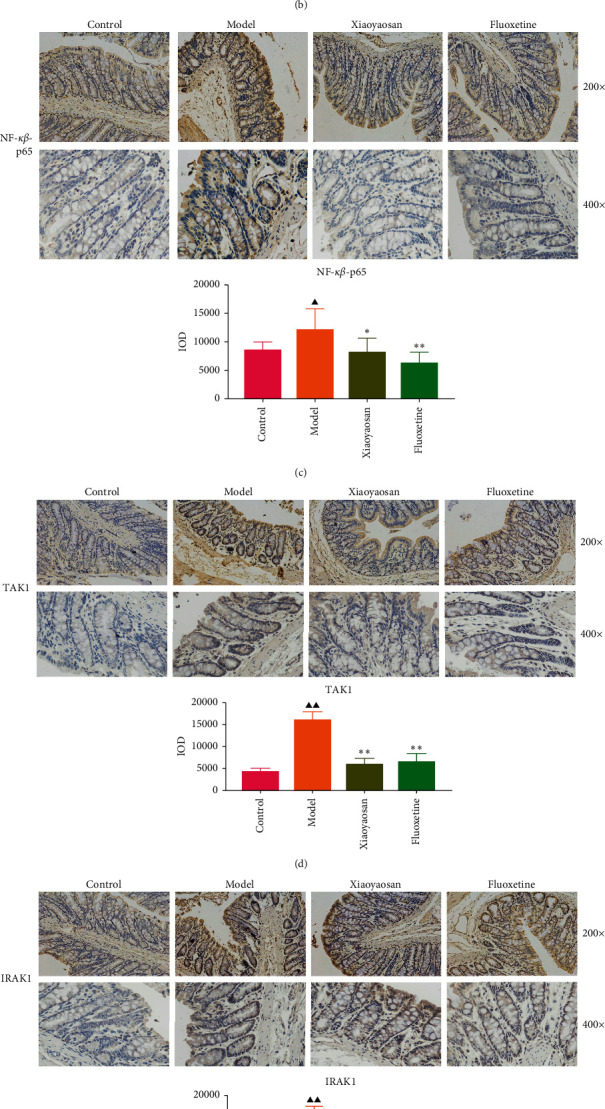
The effect of Xiaoyaosan on the immunohistochemistry of TLR4, MyD88, NF-*κ*B-p65, TAK1, IRAK1, and TRAF6 in the colon. (a)–(f) Immunohistochemical analysis of TLR4, MyD88, NF-*κ*B-p65, TAK1, IRAK1, and TRAF6 expression in colonic tissue (×200 and ×400 magnification) and quantification of TLR4, MyD88, NF-*κ*B-p65, TAK1, IRAK1, and TRAF6 immunohistochemical staining. Bars represent the means ± SD of 5 rats per group. ^▲^*p* < 0.05 and ^▲▲^*p* < 0.01 vs. the control group; ^*∗*^*p* < 0.05 and ^*∗∗*^*p* < 0.01 vs. the model group. TLR4, Toll-like receptor 4; MyD88, myeloid differentiation primary response protein; IRAK1, interleukin 1 receptor-associated kinase1; TAK1, transforming growth factor beta-activated kinase 1; TRAF6, tumor necrosis factor receptor-associated factor 6; NF-*κ*B-p65, nuclear factor kappa beta-p65.

Immunohistochemical analysis (Figure 6) showed that the colon cells of the control group were well arranged, and relatively few small brown granules, representing TLR4, MyD88, NF-*κ*B-p65, TAK1, IRAK1, and TRAF6 staining, were evident in the cytoplasm. However, in the model group, the colon cell arrangement was irregular, the number of small brown granules representing the above proteins was significantly increased, and the staining was more intense. After 3 weeks of intragastric administration of XYS and fluoxetine, the expression of TLR4, MyD88, NF-*κ*B-p65, TAK1, IRAK1, and TRAF6 was significantly reduced in both treatment groups. Further quantitative analysis using IOD showed that the levels of TLR4 (*p* < 0.01, *F* = 4.565), MyD88 (*p* < 0.01, *F* = 9.785), NF-*κ*B-p65 (*p* < 0.05, *F* = 6.486), TAK1 (*p* < 0.01, *F* = 29.217), IRAK1 (*p* < 0.01, *F* = 4.835), and TRAF6 (*p* < 0.01, *F* = 16.399) were significantly increased in the model group; however, XYS or fluoxetine treatment reduced the levels of TLR4 (XYS: *p* < 0.05, fluoxetine: *p* < 0.01, *F* = 4.565), MYD88 (all *p* < 0.01, *F* = 9.785), NF-*κ*B-p65 (XYS: *p* < 0.05, fluoxetine: *p* < 0.01, *F* = 6.486), TAK1 (all *p* < 0.01, *F* = 29.217), and TRAF6 (all *p* < 0.01, *F* = 16.399); the level of IRAK1 was also decreased, although not significantly (Figure 6).

### 3.5. The Effects of XYS on IL-1*β*, IL-6, and TNF-*α* Levels in Serum and Colon

**Figure 7:**
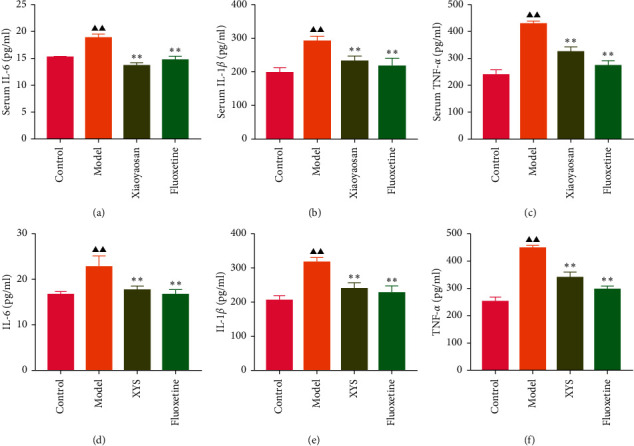
Xiaoyaosan (XYS) treatment reduced serum (a)–(c) and colon (d)–(f) levels of inflammatory factors (IL-6, IL-1*β*, and TNF-*α*). Bars represent the means ± SD of 5 rats per group. ^▲^*p* < 0.05 vs. the control group; ^▲▲^*p* < 0.01 vs. the control group; ^*∗*^*p* < 0.05 vs. the model group; ^*∗∗*^*p* < 0.01 vs. the model group. TNF-*α*, tumor necrosis factor-*α*; IL-1*β*, interleukin 1 beta; IL-6, interleukin 6.

The levels of IL-1*β*, IL-6, and TNF-*α* were determined by ELISA. Compared with the control group, the serum levels of IL-1*β*, IL-6, and TNF-*α* in the model group displayed a significant increase (*p* < 0.01; *F* = 5.007, *F* = 4.108, and *F* = 9.509, respectively). After 3 weeks of XYS treatment, the levels of IL-1*β*, IL-6, and TNF-*α* had decreased significantly (*p* < 0.01; *F* = 5.007, *F* = 4.108, and *F* = 9.509, respectively) (Figures 7(a)–7(c)). The colon levels of IL-6, IL-1*β*, and TNF-*α* in the depression model group was significantly increased (*p* < 0.01; *F* = 19.265, *F* = 38.3, and *F* = 57.968, respectively) compared with the control group. However, XYS and fluoxetine treatment significantly reduced the secretion of IL-6, IL-1*β*, and TNF-*α* (*p* < 0.01; *F* = 19.265, *F* = 38.3, and *F* = 57.968, respectively) (Figures 7(d)–7(f)). The ELISA results showed that XYS could significantly reduce the levels of inflammatory factors in depressed rats.

### 3.6. The Effects of XYS on the mRNA and Protein Expression of TLR4, MyD88, NF-*κ*B-p65, TAK1, IRAK1, TRAF6, NLRP3, Caspase-1, and ASC in the Rat Colon

**Figure 8:**
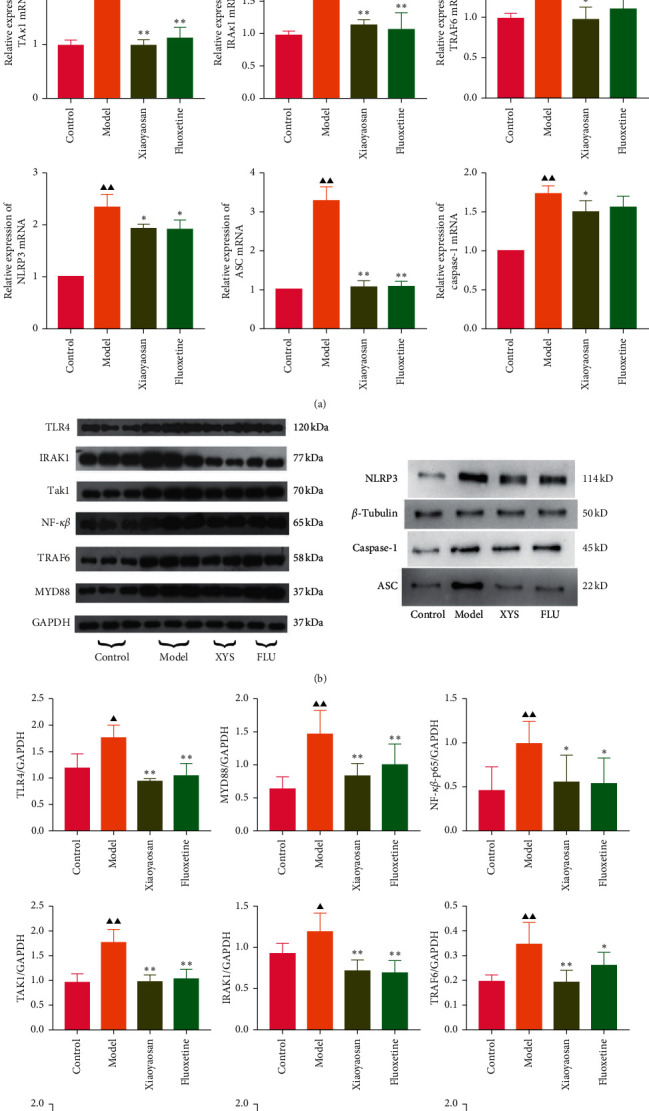
The effects of XYS on the mRNA and protein expression of TLR4, MyD88, NF-*κ*B-p65, TAK1, IRAK1, TRAF6, NLRP3, caspase-1, and ASC in the rat colon. (a) The effects of XYS on the mRNA expression levels of *Tlr4, Myd88, NF-κB-p65, Tak1, Irak1, Traf6, Nlrp3,* caspase-1, and *Asc* in the rat colon. For *Tlr4, Myd88, NF-κB-p65, Tak1, Irak1,* and *Traf6,* data are expressed as the means ± SD of 5 rats per group. ^*∗*^*p* < 0.05 and ^*∗∗*^*p* < 0.01 vs. the model group; ^▲^*p* < 0.05 and ^▲▲^*p* < 0.01 vs. the control group. For *Nlrp3*, caspase-1, and *Asc*, data are expressed as the means ± SD of 3 rats per group. ^*∗*^*p* < 0.05 and ^*∗∗*^*p* < 0.01 vs. the model group; ^▲^*p* < 0.05 and ^▲▲^*p* < 0.01 vs. the control group. (b)-(c) Semiquantitative Western blot analysis of TLR4, MyD88, NF-*κ*B-p65, TAK1, IRAK1, and TRAF6 expression in colonic tissue; data are expressed as the means ± SD of 5 rats per group. ^▲^*p* < 0.05 and ^▲▲^*p* < 0.01 vs. the control group; ^*∗*^*p* < 0.05 and ^*∗∗*^*p* < 0.01 vs. the model group. Semiquantitative Western blot analysis of NLRP3, ASC, and caspase-1 expression in colonic tissue; data are expressed as the means ± SD of 3 rats per group. ^▲^*p* < 0.05 and ^▲▲^*p* < 0.01 vs. the control group; ^*∗*^*p* < 0.05 and ^*∗∗*^*p* < 0.01 vs. the model group. TLR4, Toll-like receptor 4; MyD88, myeloid differentiation primary response protein; IRAK1, interleukin 1 receptor-associated kinase 1; TAK1, transforming growth factor beta-activated kinase 1; TRAF6, tumor necrosis factor receptor-associated factor 6; NF-*κ*B-p65, nuclear factor kappa beta-p65; NLRP3, NOD-like receptor protein 3; ASC, apoptosis-associated speck-like protein; XYS, Xiaoyaosan; FLU, fluoxetine.

The relative expression levels of genes coding for TLR4, MyD88, NF-*κ*B-p65, TAK1, IRAK1, TRAF6, NLRP3, caspase-1, and ASC were detected by qPCR. In this study, the mRNA levels of *Tlr4* (*p* < 0.01, *F* = 25.762), *Myd88* (*p* < 0.01, *F* = 13.352), NF-*κ*B-p65 (*p* < 0.01, *F* = 42.469), *Tak1* (*p* < 0.01, *F* = 35.626), *Irak1* (*p* < 0.01, *F* = 29.723), *Traf6* (*p* < 0.01, *F* = 4.840), *Nlrp3* (*p* < 0.01, *F* = 36.286), caspase-1 (*p* < 0.01, *F* = 22.024), and *Asc* (*p* < 0.01, *F* = 84.990) were increased in the model group compared with the control group. However, compared with the model group, XYS treatment resulted in decreased levels of *Tlr4* (*p* < 0.01, *F* = 25.762), *Myd88* (*p* < 0.01, *F* = 13.352), NF-*κ*B-p65 (*p* < 0.01, *F* = 42.469), *Tak1* (*p* < 0.01, *F* = 35.626), *Irak1* (*p* < 0.01, *F* = 29.723), *Traf6* (*p* < 0.01, *F* = 4.840), *Nlrp3* (*p* < 0.01, *F* = 36.286), caspase-1 (*p* < 0.05, *F* = 22.024), and *Asc* (*p* < 0.01, *F* = 84.990) mRNA in depressed rats (Figure 8(a)).

The protein expression levels of TLR4, MyD88, NF-*κ*B-p65, TAK1, IRAK1, and TRAF6 were analyzed by Western blotting. Compared with the control group, the expression levels of TLR4 (*F* = 13.954), MyD88 (*F* = 8.393), NF-*κ*B-p65 (*F* = 3.617), TAK1 (*F* = 18.689), and TRAF6 (*F* = 7.184) were significantly increased (all *p* < 0.01) in the model group, as was that of IRAK1 (*p* < 0.05, *F* = 10.157). However, when compared with the model group, the levels of TLR4, MYD88, NF-*κ*B-p65, TAK1, and TRAF6 were significantly decreased in the XYS treatment group (*p* < 0.01), as was that of NF-*κ*B-p65 (*p* < 0.05). The protein levels of TLR4, TAK1, IRAK1 (all *p* < 0.01), MYD88, NF-*κ*B-p65, and TRAF6 (all *p* < 0.05) were significantly decreased in the fluoxetine treatment group compared with the model group.

The expression levels of NLRP3, ASC, and caspase-1 were also assessed by Western blotting. Compared with the control group, the expression levels of NLRP3, caspase-1 (both *p* < 0.01), and ASC (*p* < 0.05) were significantly increased in the model group. However, compared with the model group, the protein expression levels of the three proteins were significantly decreased (NLRP3 and ASC: *p* < 0.01; caspase-1: *p* < 0.05) in the XYS treatment group. Similarly, the levels of NLRP3, ASC, and caspase-1 were all significantly decreased (all *p* < 0.01) in the fluoxetine group compared with those in the model group (NLRP3: *F* = 15.779; ASC: *F* = 11.012; caspase-1: *F* = 8.463). Combined, the Western blotting results showed that XYS could significantly reduce the levels of proteins in the TLR4 signaling pathway as well as those of NLRP3 inflammasome-related proteins (Figures 8(b) and 8(c)).

## 4. Discussion

To the best of our knowledge, this is the first study to report on the mechanism underlying the ameliorating effects of XYS on depression from the perspective of gastrointestinal inflammation based on the TLR4/NLRP3 inflammasome signaling pathway. This study offers new insights into the antidepressive mechanism of action of XYS in vivo and provides a basis for the clinical use of XYS in the treatment of depression.

In this study, we used colonic tissue to study depression and investigated key factors involved in the TLR4/NLRP3 inflammasome signaling pathway by H&E staining, immunohistochemistry, qPCR, and Western blotting. Our results showed that the expression levels of TLR4, MyD88, NF-*κ*B-p65, TAK1, IRAK1, TRAF6, inflammasome-related NLRP3, ASC, and caspase-1 were all increased in rats of the depression model group. XYS and fluoxetine treatment led to the downregulation of these factors and subsequently also the levels of inflammatory cytokines such as IL-6, IL-1*β*, and TNF-*α*. The results also showed that chronic restraint stress can lead to the TLR4 signaling pathway/NLRP3 inflammasome-induced activation of inflammatory responses, leading to an increase in the levels of inflammatory factors.

Depression may represent a chronic low-level inflammatory response involving neuroimmune-endocrine factors. Recent well-designed studies have confirmed that adverse life events, chronic stress, and depression can increase the likelihood of recurrence in patients with static inflammatory bowel disease (IBD). Experimental stress studies using animal models of colitis increasingly support this evidence. With the development of neuroimmunology, it is increasingly possible to unravel the mechanisms underlying how the nervous system affects immune function at the systemic and intestinal mucosal levels. The latest data show that chronic stress causes gastrointestinal immune inflammation [23]. Chronic stress may not only lead to intestinal barrier damage, immune cells activated, and to produce proinflammatory cytokines but also by the neural adrenergic nerve immune pathway to act on molecules expression on immune cells, such as the Toll receptor to produce inflammation. These inflammatory cytokines can not only affect the enteric nervous-smooth muscle, increased visceral sensitivity, intestinal motility, and mucosal secretion but also act on the central nervous system to produce changes in structure and function of mental symptoms of depression [24].

A large number of studies have shown that there is a complex interaction between the immune system and the CNS [25]. The immune system can affect the structure and function of the CNS, which can lead to changes in mood and behavior. The mechanisms involved in this interaction remain unclear. Nevertheless, inflammatory responses are considered to be an important link in the interaction between the immune system and the body, and there is substantial evidence to support that inflammatory mediators contribute to the interaction between the immune system and the CNS [26–29]. It is now known that inflammatory signals can be transmitted into the brain through humoral and neural pathways, and that changes in the brain immune environment and the production of inflammatory mediators are involved in the regulation of brain functions related to behavior, such as neurotransmitter metabolism, neuroendocrine function, and synaptic plasticity. Additionally, the intestinal mucosal immune response is also involved in the regulation of immune and inflammatory responses [30]. A “brain–gut axis” dialog pathway exists between the peripheral immune system and the CNS and is closely related to the occurrence and development of CNS diseases. The brain–gut axis-mediated transmission of inflammatory signals from the periphery to the CNS is considered to be an important link between CNS diseases with peripheral inflammation. The transmission of peripheral inflammatory signals to the CNS can lead to changes in neuronal signal transmission and neural circuit function. Under long-term chronic stimulation, alterations in mood and behavior can occur [31].

In humans, pattern recognition receptors play a critical role in innate immunity. The TLR4 signaling pathway and the NLRP3 inflammasome are closely related to depression and inflammatory bowel disease. How does the TLR4/NLRP3 inflammasome signaling pathway induce the inflammation-associated immune response in depression? TLR4 activation is induced by chronic restraint stress. TLR4 can recognize and bind lipopolysaccharide (LPS), leading to the activation of the death domain of MyD88, which recruits and activates the linker proteins IRAK4, IRAK1, and TRAF6, among others. TRAF6 can form a complex with UBC1/3UEV1A and function as an E3 ubiquitin ligase that activates TAK1 at the membrane; TAK1 then activates the downstream inhibitory I*κ*B kinase and mitogen-activated protein kinase (MAPK) pathways, ultimately leading to the activation of an inflammatory reaction [32, 33]. NLRP3 binds to ASC, resulting in the enhanced production of procaspase-1. Procaspase-1 is subsequently converted to activated caspase-1, which further promotes the secretion of IL-1*β*, thereby generating a corresponding inflammatory response and promoting the pathogenesis of depression [11, 12]. TLR4 activation leads to the release of NF-*κ*B, its translocation into the nucleus, and activation of the NLRP3 inflammasome.

TLR4/NLRP3 plays key roles in regulating intestinal homeostasis, maintaining intestinal epithelial barrier integrity, and reducing mortality during experimental colitis and can also affect the composition of the intestinal biota [34–37]. How would the peripheral inflammatory response induced by TLR4/NLRP3 in the colon affect CNS behavior? The effects of TLR4/NLRP3-mediated peripheral immune activation in the colon on behavior are mainly mediated via three mechanisms: (1) the release of proinflammatory cytokines at the periphery can directly increase the signal transduction of neuroregulatory cytokines through the blood-brain barrier (BBB) via “leakage” through the circumventricular organs; (2) activated immune cells can also directly cross the BBB as a form of neuroimmune signal transduction; and (3) peripheral cytokines can stimulate afferent pathways, such as the vagus nerve, and promote behavioral changes via neurological mechanisms [38]. Immune inflammatory response is one of the pathogenesis of depression. The cytokines produced by it can induce depression mainly through the following ways after passing through the blood-brain barrier: intensifying the brain immune cell response; activating the neuroendocrine axis; inhibiting monoamine neurotransmitters; and changing the structure and function of brain regions related to emotion regulation to induce depression. [39].

In summary, chronic restraint stress can cause a gastrointestinal immune response, which is primarily mediated through the TLR4/NLRP3 inflammasome signaling pathway. Activation of this pathway may represent a common pathological process underlying the development of depression and gastrointestinal disease. Inflammatory cytokines are markers of immune system activation and also mediators of the CNS and immune system activity. Inflammatory cytokines passing through blood-brain barrier may intensify the brain immune cell response; activate the neuroendocrine axis; inhibit monoamine neurotransmitters; and change the structure and function of brain regions related to emotion regulation and induce depression.

Studies have shown that tetramethylpyrazine (TMP) can reverse increases in the levels of inflammatory cytokines such as TNF-*α*, IL-1*β*, and IL-6 and inhibit the upregulation of the levels of TLR4/p38/NF-*κ*B/NLRP3 signaling-associated proteins in the prefrontal cortex and hippocampus [40]. These results suggest that TMP may exert a potential antidepressive-like effect in a model of CUMS-induced depression. Using the same model, a different study [41] showed that Chaihu Shugan San treatment can reduce the serum levels of IL-1*β*. This formula can also inhibit hepatic and prefrontal cortical inflammatory responses by suppressing the TLR4/MyD88/NF-*κ*B pathway and activating the NLRP3 inflammasome and can also ameliorate depressive-like behaviors by inhibiting the liver-brain inflammation axis. He et al. [42] reported that muscone can ameliorate LPS-induced depressive-like behaviors by repressing neuroinflammation in the prefrontal cortex of mice through suppressing microglial activation and the production of inflammatory cytokines via the TLR4 pathway. Overall, our results are consistent with those of the abovedescribed studies. Inhibiting the TLR4/NLRP3 inflammasome signaling pathway and reducing inflammatory cytokine levels are commonly used as a means of exploring whether drugs can suppress depressive-like behavior. However, the above studies are based on the prefrontal cortex, hippocampus, and liver-brain axis, among others, whereas we investigated the mechanism of action of XYS in reducing depressive-like behaviors with respect to an intestinal inflammatory immune response.

In TCM theory, dysfunction of the spleen and stomach can lead to dysfunction of transportation, lack of metaplasia of Qi and blood, and dysfunction of Qi elevation and descent, thus leading to depression. In the 1990s, dysfunction of Qi caused by the spleen and stomach dysfunction was proposed to be the main pathogenic mechanism of depression [43]. TCM is commonly used to regulate the spleen and stomach in the treatment of depression. Shi et al. [44] used a TCM approach to regulate the spleen and stomach to treat depression and showed that the short-term efficacy of this method was similar to that of fluoxetine hydrochloride capsules. XYS has been demonstrated to effectively interfere with CNP/NPR-B signaling pathway activity in the rectum of depressed rats [45]. XYS can improve the gastrointestinal and thus help with treating depression, through its ability to regulate effects at multiple levels, via multiple channels, and directed at multiple targets. Gao et al. [46] identified saikosaponin A, saikosaponin C, saikosaponin D, ferulic acid, Z-ligustrazine, atrazine enol I, atrazine acetenol II, atrazine acetenol III, paeoniflorin, leucaenanthoside, glycyrrhizic acid, and chlorpyruvic acid as the main antidepressive components of XYS, reflecting the multicomponent, multitarget, and multichannel characteristics of TCM.

**Figure 9:**
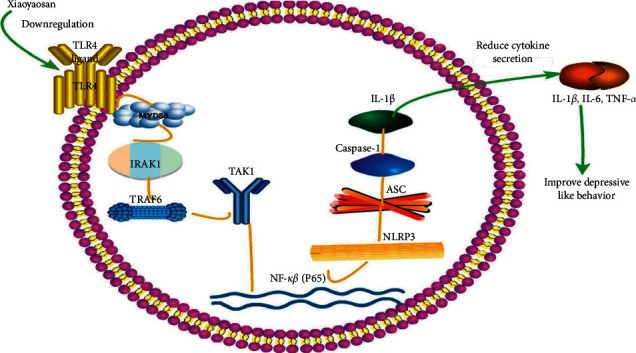
Xiaoyaosan exerts its antidepressive effects in rats with chronic restraint-induced stress via regulating the activation of the immunoinflammatory response induced by the TLR4/NLRP3 inflammasome signaling pathway in the colon. Xiaoyaosan treatment reduced the expression levels of TLR4, MyD88, NF-*κ*B-p65, TAK1, IRAK1, TRAF6, NLRP3, ASC, and caspase-1 in colonic tissue of depressed rats; reduced the serum levels of the inflammatory factors IL-6, IL-1*β*, and TNF-*α*; and ameliorated colonic inflammation. TLR4, Toll-like receptor 4; MyD88, myeloid differentiation primary response protein; IRAK1, interleukin 1 receptor-associated kinase 1; TAK1, transforming growth factor beta-activated kinase 1; TRAF6, tumor necrosis factor receptor-associated factor 6; NF-*κ*B-p65, nuclear factor kappa beta-p65. NLRP3, NOD-like receptor protein 3; ASC, apoptosis-associated speck-like protein.

In conclusion, the results of this study demonstrated that XYS can improve depressive-like behavior in rats by suppressing the activation of the TLR4/NLRP3 inflammasome signaling pathway, thereby inhibiting immunoinflammatory activation and reducing the levels of inflammatory cytokines in the colon. The observed improvement in depressive symptoms may have resulted from the downregulation of the levels of TLR4, MyD88, NF-*κ*B-p65, TAK1, IRAK1, TRAF6, and NLRP3 inflammasome-related NLRP3, ASC, and caspase-1 proteins, leading to the subsequent downregulation of the levels of the inflammatory cytokines IL-6, IL-1*β*, and TNF-*α* (Figure 9).

## 5. Conclusions

XYS can improve depressive-like behavior by suppressing the TLR4/NLRP3 inflammasome signaling pathway, thereby inhibiting the activation of immunoinflammatory responses and, consequently, reducing the levels of inflammatory cytokines in the colon. This study offers new insights into the mechanism underlying the mode of action of XYS in vivo and provides a basis for the clinical use of XYS as an antidepressant.

## Figures and Tables

**Table 1:** Composition of Xiaoyaosan.

English name	Chinese name	Latin name	Scientific name	Part used
Chinese thorowax root	Chai Hu	Radix Bupleuri	*Bupleurum chinense* DC	Root
Chinese angelica root	Dang Gui	Radix Angelicae Sinensis	*Angelica sinensis* (Oliv.) Diels	Root
White peony root	Bai Shao	Radix Paeoniae Alba	*Paeonia lactiflora* Pall.	Root
Large head atractylodes rhizome	Bai Zhu	Rhizoma Atractylodes Macrocephalae	*Atractylodes macrocephala* Koidz.	Rhizoma
Poria cocos	Fu Ling	Poria	*Poria cocos* (Schw.) Wolf	Sclerotia
Fresh ginger rhizoma	Sheng Jiang	Zingiberis Recens	*Zingiber officinale* Rosc.	Rhizoma
Peppermint	Bo He	Herba Menthae	*Mentha haplocalyx* Briq.	Stems and leaves
Liquorice root	Gan Cao	Radix Glycyrrhizae	*Glycyrrhiza uralensis* Fisch.	Root

**Table 2:** Primer sequences used for RT-qPCR.

Gene	Forward primer (5′–3′)	Reverse primer (5′–3′)
Irak1	TCCTAACAGAGGTGGAACAG	GATCCTCTAAGGAGCCATTG
Myd88	GTCGCATGGTGGTGGTTGTT	GGATCAGTCGCTTCTGTTGG
NF-*κ*B-p65	AGCTATAACTCGCCTGGTGA	ATGTCCGCAATGGAGGAGAA
Tak1	GTATGGAGCCTGCTTGAATC	GGCATGAGCAGCAGTGTAAT
Tlr4	CAGAGCCGTTGGTGTATCTT	AGGCGATACAATTCGACCTG
Traf6	GAAGCACAGCAGTGTAATGG	CAGGTCTGCCTGTGTAGAAT
Gapdh	ACCACAGTCCATGCCATCAC	TCCACCACCCTGTTGCTGTA
Nlrp3	CGAGGTCTCTTCTCAAGTCT	CCAACCACAGTCTCTGAATG
Asc	GCACAGCCAGAACAGAACAT	AACTGCCTGGTACTGTCCTT
Casp1	CCTGTGCGATCATGTCACTA	AGCTGATGGACCTGACTGAA

## Data Availability

The data used to support the findings of this study are available from the first author (zhuhuizheng@jnu.edu.cn) upon request.
